# Human Pathogenic Monkeypox Disease Recognition Using Q-Learning Approach

**DOI:** 10.3390/diagnostics13081491

**Published:** 2023-04-20

**Authors:** Malathi Velu, Rajesh Kumar Dhanaraj, Balamurugan Balusamy, Seifedine Kadry, Yang Yu, Ahmed Nadeem, Hafiz Tayyab Rauf

**Affiliations:** 1 School of Computer Science and Engineering, Panimalar Engineering College, Poonamallee, Chennai 600123, India; 2 School of Computing Science and Engineering, Galgotias University, Greater Noida 203201, India; 3 Associate Dean-Student Engagement, Shiv Nadar Institution of Eminence, Delhi-National Capital Region (NCR), Gautam Buddha Nagar 201314, India; 4 Department of Applied Data Science, Noroff University College, 4612 Kristiansand, Norway; 5 Artificial Intelligence Research Center (AIRC), Ajman University, Ajman P.O. Box 346, United Arab Emirates; 6 Department of Electrical and Computer Engineering, Lebanese American University, Byblos 13-5053, Lebanon; 7 Centre for Infrastructure Engineering and Safety (CIES), The University of New South Wales, Sydney, NSW 2052, Australia; 8 Department of Pharmacology and Toxicology, College of Pharmacy, King Saud University, P.O. Box 2455, Riyadh 11451, Saudi Arabia; 9 Centre for Smart Systems, A.I. and Cybersecurity, Staffordshire University, Stoke-on-Trent ST4 2DE, UK

**Keywords:** deep Q-learning network, policy gradient, Actor–Critic, optimization, monkeypox, deep convolutional neural network

## Abstract

While the world is working quietly to repair the damage caused by COVID-19’s widespread transmission, the monkeypox virus threatens to become a global pandemic. There are several nations that report new monkeypox cases daily, despite the virus being less deadly and contagious than COVID-19. Monkeypox disease may be detected using artificial intelligence techniques. This paper suggests two strategies for improving monkeypox image classification precision. Based on reinforcement learning and parameter optimization for multi-layer neural networks, the suggested approaches are based on feature extraction and classification: the Q-learning algorithm determines the rate at which an act occurs in a particular state; Malneural networks are binary hybrid algorithms that improve the parameters of neural networks. The algorithms are evaluated using an openly available dataset. In order to analyze the proposed optimization feature selection for monkeypox classification, interpretation criteria were utilized. In order to evaluate the efficiency, significance, and robustness of the suggested algorithms, a series of numerical tests were conducted. There were 95% precision, 95% recall, and 96% f1 scores for monkeypox disease. As compared to traditional learning methods, this method has a higher accuracy value. The overall macro average was around 0.95, and the overall weighted average was around 0.96. When compared to the benchmark algorithms, DDQN, Policy Gradient, and Actor–Critic, the Malneural network had the highest accuracy (around 0.985). In comparison with traditional methods, the proposed methods were found to be more effective. Clinicians can use this proposal to treat monkeypox patients and administration agencies can use it to observe the origin and current status of the disease.

## 1. Introduction

Monkeypox reports for 2022 indicate yet another worldwide virus following the COVID-19 epidemic that shook the world in 2020 [1]. Smallpox and cowpox viruses are closely related to this one. The main carriers of the disease are rats and monkeys. However, it is also common human-to-human transmission [2]. The virus was originally found in monkeys at a laboratory in Copenhagen, Denmark, in 1958 [3]. Monkeypox was reported in the Democratic Republic of the Congo in 1970 at a time when efforts to eradicate smallpox were intensifying [4]. It is well known that the extremely infectious monkeypox virus affects a large number of residents of tropical rainforests in Central and West Africa. The infection can spread by physical communication with the diseased person. Saliva, nasal secretions, or respiratory droplets can spread an infection [5]. Animal bites might also spread it. A variety of symptoms, such as fever, aches, fatigue, and red skin bumps over time, are experienced by patients with monkeypox [6]. Monkeypox is not closely as spreadable as COVID-19 has been, despite the fact that the count of cases that have been documented is increasing [7]. In the year 1990, only fifty persons in West and Central Africa had monkeypox [8]. However, 5000 documented incidents were reported by the year 2020. It was previously thought that monkeypox outbreaks only occurred in Africa, but in 2022, a number of non-African countries, including Europe and the U.S., stated the identification of monkeypox infections among individuals. As a result, there is increasing dread and alarm among the general public, and many people are expressing their worries online. There is currently no cure for the monkeypox virus, according to the CDC’s recommendations [9]. Nevertheless, vaccination offers a potent defence against the monkeypox virus. Despite the fact that there are FDA-approved vaccines against the monkeypox virus, no individuals have received them in the U.S. In other nations, monkeypox treatment often entails immunization against smallpox [10]. The medical history of the patient and the unique characteristics of the skin lesions themselves are used to identify monkeypox. The gold standard for determining if a skin lesion is viral in nature is electron microscopy testing. Additionally, polymerase chain reaction (PCR) [11], a method frequently used to detect COVID-19 patients [12], can be used to confirm the monkeypox virus. Machine Learning (ML) is a young subject of artificial intelligence [13] that has shown considerable promise in a range of applications, including helping people make decisions, industrial applications [14], medical imaging, and sickness detection [15]. ML-enabled imaging systems have been discovered by medical practitioners to be important tools for making rapid, accurate, and safe judgments. Medical experts have discovered that the safe, precise, and rapid imaging solutions enabled by ML are priceless tools for making wise choices. For instance, the authors of [16] developed CAD systems based on fuzzy logic for the goal of diagnosing breast cancer. Fuzzy logic is preferable to classical ML because it can speed up computing processes while imitating the cognitive strategy of an expert radiologist. If the user enters details such as contour, density, and shape, the cancer detection algorithm will produce a result depending on the approach they choose [17]. The authors evaluated ten kinds of DL models and attained 99.1% accuracy for 108 patients with COVID-19 and 86 non-COVID-19 patients [18]. The authors created an improved inception model with the help of 453 CT scan images, increasing its accuracy to 73.1% [19]. Skin conditions such as psoriasis, melanoma, lupus, and chickenpox are just a few that may be identified using the CNN recommended [20]. They showed that using picture analysis and an already-built VGGNet, skin disease can be identified 71% of the time. The suggested method performed the best, with an accuracy of roughly 78%. The authors created a technique for identifying skin disorders using mobile nets and cell phones [21]. They claimed that when detecting people with chickenpox symptoms, their accuracy was 94.4%. Currently, there is little research that indicates the potential for the use of ML techniques to image processing-based monkeypox diagnosis. The absence of a publicly accessible data repository for training and testing purposes was caused by the virus’s recent significant introduction in many countries, which made it impossible to establish a model for the identification of monkeypox.

In light of these possibilities, it is required to create a fresh strategy for accurately identifying monkeypox photos in order to close this gap. In order to fill it, this study suggests two novel techniques that will enhance both the performance of classifiers and the selection of the optimal collection of features. The Al-Biruni Earth radius (BER), the sine cosine algorithm (SCA), and particle swarm optimization are the foundations for these two techniques (PSO). A series of tests were carried out to demonstrate the efficacy of the suggested algorithms, and the outcomes are contrasted with those of rival feature selection and parameter optimization techniques. The suggested algorithms’ stability was tested statistically, and the findings supported the intended result.

The major contribution of the work is as follows:

1. The detailed survey relevant to the classification of monkeypox diseases was carried out. The authors’ contribution, limitations, and future scope are discussed;
2. The proposed work is developed to recognize the Monkeypox Virus with respect to four classes;
3. The performance of the model can be measured with the help of evaluation metrics, namely, AUC, CA, F1, precision, and Recall. The DQN approach achieves a classification accuracy (C.A.) of 0.975;
4. The comparison of the proposed work with the benchmark mark algorithms, namely, DQN, DDQN, Policy Gradient, and Actor–Critic. Compared with other state-of-the-art methods, the proposed DQN outperforms others with higher accuracy and AUC.

The organization of the paper is as follows: Section 2 explains the literature review and the main contribution of the work; Section 3 describes the proposed work, and the subsection includes the dataset, pre-processing, and reinforcement method; Section 4 describes the results and discussion of the proposed method; and Section 5 explains the conclusion and future work.

## 2. Related Works

Deep learning and machine learning have shown themselves to be quite helpful in the diagnosis and treatment of medical conditions. To forecast illnesses, researchers have developed systems using ML and DL. For Alzheimer’s disease, there is presently no accurate diagnostic procedure. The authors searched EEG epochs for characteristics that would distinguish Alzheimer’s patients from controls with the help of an ML technique called the Support Vector Machine (SVM) [22]. The accuracy of the research was good since it took into account how each patient’s diagnosis was made.

One of the top five leading causes of transience in the world nowadays is heart disease. One of the biggest problems in medical detection is predicting cardiovascular disease. Machine learning has been shown to be capable of sifting information generated by the healthcare sector to find relevant information. A number of studies have just begun to scratch the surface of the potential applications of ML to heart disease prediction. The authors of [23] proposed a technique to advance cardiovascular disease identification by identifying key variables using ML techniques. The prediction model examined a diversity of feature arrangements and well-known classification techniques [24]. Parkinson’s disease (P.D.) diagnosis is frequently reached following extensive medical evaluation and examination of clinical indications [25]. To finish this assessment, a range of motor symptoms must frequently be defined. However, conventional diagnostic techniques rely on the subjective estimation of gestures that could be challenging to spot [26]. By using machine learning algorithms, we may be able to identify relevant traits that are underused in the medical analysis of Parkinson’s disease and which may be used to identify P.D. Liver disease is prevalent in medical settings and is linked to a higher risk of death (FLD). The ability to progress a practical strategy for anticipation, initial analysis, and treatment is provided by early analysis of FLD patients. The authors proposed a machine learning system to forecast the beginning and course of the illness to help with the identification of at-risk people, the diagnosis itself, and FLD prevention and care. For the purpose of predicting FLD, a number of classification models, including logistic regression (L.R.), random forest (R.F.), I Bayes (N.B.), and an artificial neural network (ANN), have been created. The effectiveness of the four models was evaluated using the receiver operating characteristic curve area (ROC). Four categorization algorithms that accurately diagnose fatty liver disease were created and researched by the authors of [27]. However, the R.F. performed better as compared with the classification methods. The help of a random forest model in the clinical setting may be advantageous for the early treatment of patients’ liver wellness.

A severe danger to health and well-being, chronic kidney disease (CKD), affects an alarmingly rising percentage of people worldwide. Early-stage CKD frequently has no symptoms; hence, its presence is frequently disregarded. The CKD-slowing medication works best when it is administered with patients’ diagnoses. ML models’ quick and precise detection skills can help therapists achieve this goal in a big way. The authors suggested an ML approach for diagnosing CKD [28]. The machine learning repository at UCI provided the CKD data repository, which is severely biased by missing values [29]. For a variety of reasons, patients could forget or were unable to give some metrics. As a result, it is typical for data gaps to be discovered in clinical practice. When the missing data were included, six ML algorithms were utilized to create the models. As compared with other models, the R.F. model’s diagnostic accuracy was the greatest (99.75%). It was suggested to utilize a hybrid model that included logistic regression and a random forest using a perceptron after examining the flaws of the earlier models, reaching an accuracy of 99.83%.

The authors suggested a ground-breaking ML method to accurately detect coronary artery disease (CAD). Ten tried-and-true machine-learning methods were taken into account. The use of data standardization and pre-processing increased the efficacy of these tactics [30]. The authors combined stratified ten-fold cross-validation and particle swarm optimization, a type of genetic algorithm, allowing for simultaneous optimization of feature selection and classifier parameters. The recommended technique significantly improved the accuracy of the machine learning models employed in medical and scientific research, according to experimental data. There are currently 75 nations outside of Africa where there are verified occurrences of monkeypox, making it a serious global health problem. Due to the virus’s resemblance to measles and chickenpox, it can be difficult to diagnose monkeypox early in the course of the illness. Deep learning systems have been found to be successful in automatically detecting skin lesions when given enough training data. Because monkeypox is so uncommon, there was already a knowledge gap across the globe prior to the current epidemic. In their quest to solve this puzzling problem, researchers are encouraged by the accomplishments of supervised machine learning in the identification of COVID-19. However, the scarcity of monkeypox skin photographs makes it difficult to use machine learning to identify the disease from patient skin scans. The authors provided the largest archive of images of monkeypox skin. A comprehensive image library of both healthy and unhealthy skin may now be located and used thanks to web scraping. Symptoms of measles, cowpox, chickenpox, smallpox, and monkeypox can be seen in photographs of afflicted skin [31]. The Monkeypox Skin Lesion Dataset was assembled by the authors of [32] using images of measles, chickenpox, and monkeypox skin lesions (MSLD). Most of these images originated from web pages that were open to the public. Initial approaches included a 3-fold cross-validation experiment and increased the model size with new data. The second stage involved categorizing ailments using pre-trained deep learning models, including VGG-16, ResNet50, and InceptionV3 (e.g., monkeypox).

ResNet50 achieved the highest level of overall accuracy. The authors suggested a DL model for identifying monkeypox illness that depends on picture data acquisition and execution using a modified version of VGG16 [33]. Since the data repository is created by assembling images from many open-source publications and websites, it is safer to use and distribute it for creating and installing any machine learning model. The VGG16 model with the modifications was utilized in two distinct research. According to the results of both studies, this model may successfully identify individuals who have monkeypox. The model’s capacity to anticipate and extract such properties enables the development of a greater understanding of the characteristics of the monkeypox virus. In the existing system, there is a lack of a system that can detect A.D. diseases in prior knowledge; sometimes, the model fails to converge properly. The proposed approach can reduce the convergence problem by tuning the neurons and can be used to find meaningful patterns within the data, eventually helping identify patterns for diseases other than A.D. Our conclusions are supported by our explainable artificial intelligence (XAI) techniques.

Table 1 illustrates the data analysis for the detection of monkeypox disease detection for the feature extraction model; here, the DenseNet-169 model obtained an accuracy of around 84.24%, which is higher as compared to the remaining approach. Similarly, the f1 score value is higher, around 83.83 %, as compared to the other models. Table 2 illustrates the data analysis for the detection of monkeypox disease detection for the classification process, and the reinforcement learning models are compared; the resultant value shows that the Actor–Critic learning model obtained the highest accuracy, around 89%, as compared to the other approach.

## 3. Materials and Methods

### 3.1. Dataset

**Figure 1:**
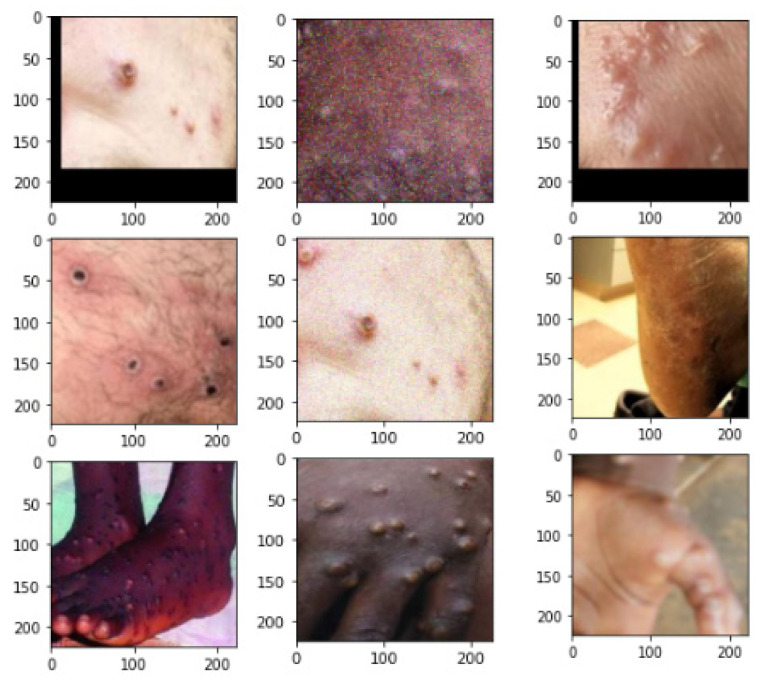
Sample dataset.

This major spate of monkeypox infections has raised concerns about public health because of its rapid expansion in over 65 nations. Timely diagnosis identification is essential to halting its rapid progression. However, significant amounts of Polymerase Chain Reaction (PCR) tests and other biochemical assays are not easily accessible [4]. Monkeypox detection from skin lesion photos using computer vision techniques may be useful in this situation. However, no such information is currently accessible. As a result, the “Monkeypox Skin Lesion Dataset (MSLD)” is made by gathering and analyzing pictures from various websites, news portals, and publicly available case reports. The “Monkeypox Image Lesion Dataset” was produced with the primary goal of separating monkeypox patients from related non-monkeypox instances. As a consequence, to produce a classifier, we introduced lesion pictures of “Chickenpox” and “Measles” to the “Monkeypox” category because of their similarity to the monkeypox rash and initial state pustules. It has a maximum of 228 photos, of which 102 are under the “Monkeypox” label and the remaining 126 are under the “Others” label, which include cases of non-monkeypox (such as chickenpox and measles) (https://www.kaggle.com/datasets/nafin59/monkeypox-skin-lesion-dataset). Figure 1 illustrates the sample raw data.

### 3.2. Data Preprocessing

#### 3.2.1. Augmented Images

Numerous image enhancement techniques, including rotation, translation, reflection, shear, hue, saturation, contrast and brightness jitter, noise, scaling, etc., were implemented with the help of MATLAB R2020a to help with the classification problem. Although Image Generator and other image augmenters make this simple to perform, the augmented images are placed in this folder to ensure the reproducibility of the results. The number of photos rose around 14-fold after enhancement. There are 1428 and 1764 photos, respectively, in the classifications “Monkeypox” and “Others”.

#### 3.2.2. Fold1

Three-fold cross-validation was carried out in order to remove the bias from the training process. With patient independence preserved, the original photos were divided about 70:10:20 into training, validation, and test sets. As per the widely accepted method of data preparation, only the training and validation sets of pictures were enhanced. Users can choose to use the folds directly or to use the original data and add other algorithms to it.

#### 3.2.3. Reinforcement Learning

The main focus of this paper is the detection of the monkeypox diseases using the Q-learning approach. This paper suggests two strategies for improving monkeypox image classification precision. Based on reinforcement learning and parameter optimization for multi-layer neural networks, the suggested approaches are based on feature extraction and classification. The Q-learning algorithm determines the rate at which an act occurs in a particular state. Malneural networks are binary hybrid algorithms that improve the parameters of neural networks.

**Figure 2:**
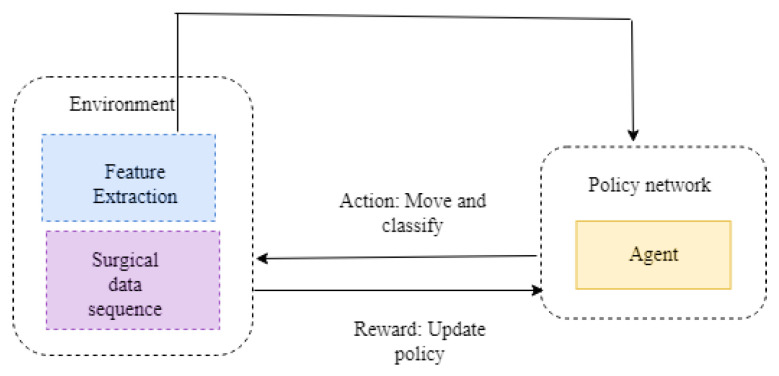
Framework of reinforcement learning.

**Figure 3:**
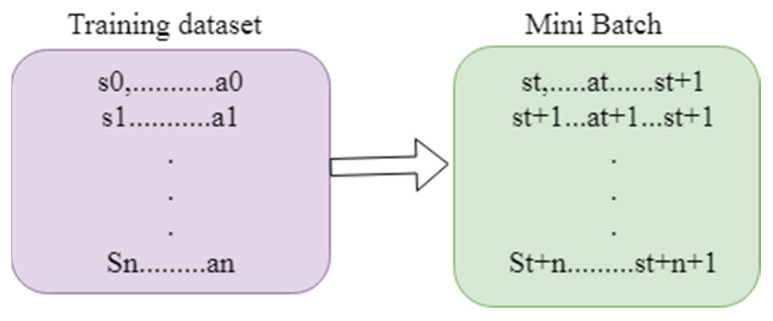
Data creation for the various model.

Reinforcement learning comes under the subgroup of machine learning. The agent read the fine-tuned policy with the help of the trial-and-error method. In real-time, this kind of approach is utilized in robotics, self-driving cars, etc. The agent learns the policy by communicating with the environment. Markov decision process is carried out by using a conditional probability distribution. Here, the future output completely depends upon the current state. The action and reward are introduced in the Markov process, called MDP. Figure 2 illustrates the framework of reinforcement learning. Figure 3 represents the data creation for the various model. In the MDP, the output obtained not only depends on the current state but also on the action that tends towards the future state of S. The trajectory distribution can be denoted as:

(1) $$W_{\pi }=\prod_{t}\pi \left(\frac{b_{t}}{q_{t}}\right)T\left(\frac{q_{t+1}}{q_{t}},b_{t}\right).$$

Here, *W* represents the length, *b_t_*, *q_t_*, *q t* + 1 are the probability of observations, and *t* represents the transition probability function. The aim of the Rl is to identify the optimal policy.

(2) $$Z=\overset{r-1}{\underset{t=0}{\sum }}\vartheta^{t}Z_{t+1}$$

The expected reward maximization can be calculated by using a formula wherein π represents the policy. The discounted expected reward can be written as Equation (4)

(3) $$E_{T}\sum_{t=0}^{r-1}r_{t+1}\to \max_{\pi };$$

(4) $$D\left(\pi \right)=E_{T\varphi }\sum_{t=0}^{T+1}\vartheta^{t}Z_{t+1}.$$

The target of the R.L. is to recognize the optimal policy:

(5) $$D\left(\pi \right)={\mathrm{Max}}_{\pi }.$$

The Bellman expectation equation can be written as Equation (5), where q represents the state, π represents the policy, and val(q(t)) represents the state value function. The transition probability can be written in Equation (7):

(6) $$\mathrm{val}\;\left(q_{t}\right)=E\left(r_{t+1}+{\gamma \;\mathrm{val}}^{\pi }\left(q_{t+1}\right)\right);$$

(7) $$\mathrm{Val}\left(q_{t}\right)=\sum_{b\in B}\pi \left(\frac{b_{t}}{s_{t}}\right)\sum_{q_{t+1\epsilon q}}T\left(\frac{q_{t+1}}{q_{t}},b_{t}\right)[Z\;\left(q_{t},q_{t+1}\right)+\vartheta {\;\mathrm{val}}^{\pi }\left(q_{t+1}\right)].$$

The above equation is known as the Bellman equation. The agent’s choice of action depends upon the optimal policy. The Bellman equation is represented as follows:

(8) $${\mathrm{val}}^{*}\left(q_{t}\right)=\max_{\mathrm{bt}}\sum_{q_{t+1}\in Q}T(q_{t+1\;/q_{t},}{\;b}_{t})[Z\left(q_{t},q_{t+1}\right)+\vartheta {\mathrm{val}}^{*}\left(q_{t+1})\right]\max Q\left(q_{t},b_{t}\right).$$

val* states the optimal value function. The quality function can be written as

(9) $$Q^{\pi }\left(q_{t},b_{t}\right)=\sum_{q_{t+1}}T\left(\frac{q_{t+1}}{q_{t}},{\;b}_{t}\right)\left[R\left(q_{t},q_{t+1}\right)+\vartheta {\;\mathrm{val}}^{\pi }\left(q_{t+1}\right)\right].$$

Figure 2 illustrates the framework of the reinforcement learning approach. Here, environment plays an important role in extracting the features and performing the surgical data sequence. The agent acts in the policy network. Based on the situation, the actions are taken. The functions of the action are to move and classify. The policy is updated from the environment to the policy network. Figure 3 illustrates the data creation for the various model, the training dataset contains s0 up to an. The mini batch contains the st to st + 1.

### 3.3. Proposed Methodology

#### 3.3.1. System Model

##### DQN

Deep Q-learning network reads the input image from the higher dimensional. Taking regression into an account, m represents the target of regression, input is (q,b), and target is (q,b). The loss function can be written as

(10) $$m\left(q_{t},b_{t}\right)=Z\left(q_{t},q_{t+1}\right)+\vartheta \max_{\mathrm{bt}+1}Q^{*}\left(q_{t},b_{t+1},\theta_{t}\right),$$

(11) $$O^{\mathrm{DQN}}=O\left(m\left(q_{t},\mathrm{bt}\right),{\;Q}^{*}\left(q_{t},b_{t},\theta_{t}\right)\right),$$

(12) $$O^{\mathrm{DQN}}={\left|\left|m\left(q_{t},\mathrm{bt}\right)-Q^{*}\left(q_{t},b_{t},\theta_{t}\right)\right|\right|}^{2\;},$$

where θ represents the vector and $\theta \in z^{\left|q\right|\left|z\right|}$ is the sample. The loss function can be minimized using

(13) $$\theta_{t+1}=\theta_{t}=\alpha_{t}\frac{\partial O^{\mathrm{DQN}}}{\partial O}.$$

**Figure 4:**
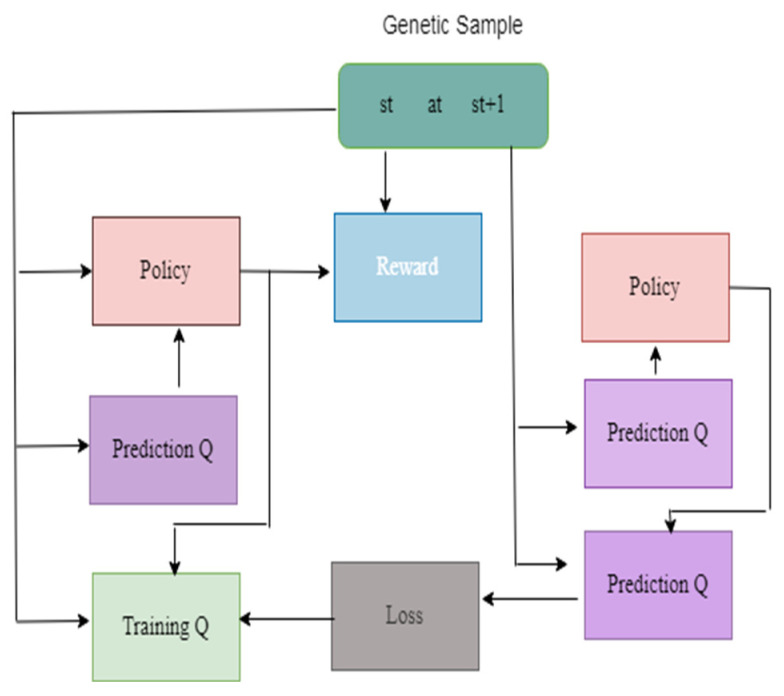
Framework of the DQN Model.

Figure 4 illustrates the framework of the DQN model. The genetic samples consist of the dataset, so it is represented as st up to st + 1. The genetic samples are connected to prediction, rewards, and policy. Based on the policy, the rewards are measured. The prediction Q helps to predict the value based on the training data. The loss value is measured and backpropagated to reduce the error value.

##### DDQN

The limitation of the deep Q-learning network is that rate of Q* enhanced due to minimum value in Equation (10). The double deep Q-learning network overcomes the overestimation of Q. It produces the better performance as compared with the deep Q-learning network.

(14) $$m1=Z\left(q_{t},q_{t+1}\right)+\vartheta Q_{1}^{*}\left(q_{t+1},{\mathrm{argmax}}_{b+1}Q_{2}^{*}\left(q_{t+1},b_{t+1};\theta_{1}\right);\theta_{1}\right)$$

(15) $$m2=Z\left(q_{t},q_{t+1}\right)+\vartheta Q_{2}^{*}\left(q_{t+1},{\mathrm{argmax}}_{b+1}Q_{2}^{*}\left(q_{t+1},b_{t+1};\theta_{1}\right);\theta_{2}\right)$$

**Figure 5:**
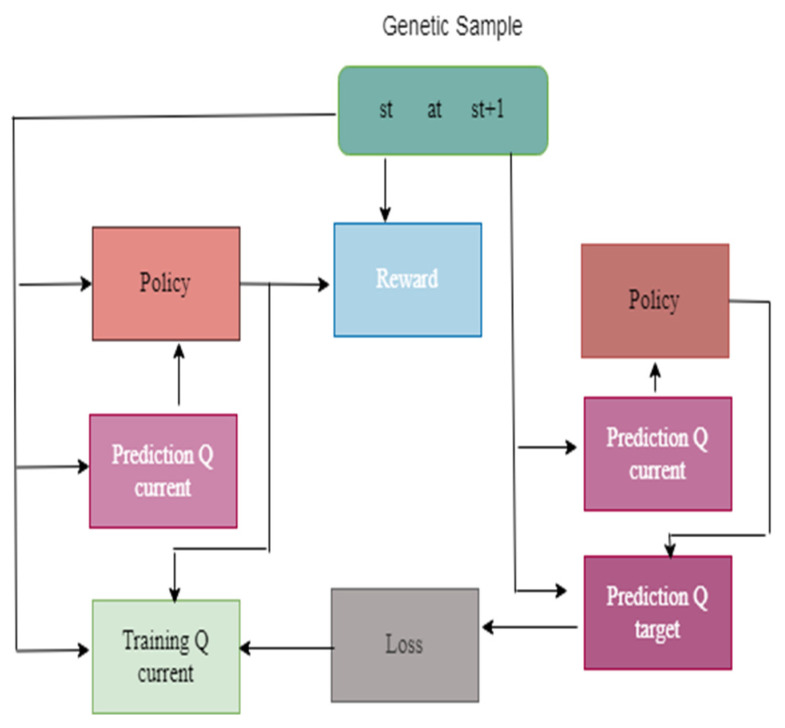
Framework of the DDQN Model.

Figure 5 illustrates the framework of DDQN model. The genetic samples consist of the dataset, so it is represented as st upto st+1. The genetic samples are connected to prediction, rewards, and policy. Based on the policy, the rewards are measured. The prediction Q helps to predicts the value based on the training data. The loss value is measured and backpropagated to reduce the error value. In this deep process, the neural network is designed in a detailed manner to predict the value.

##### Policy Gradient

The Policy Gradient DRL optimizes the objective function:

(16) $$D\left(\theta \right)=E_{T\sim \pi \theta }\sum_{t=1}\gamma^{t-1}Z\;\left(Q_{t-1},q_{t}\right)\to {max}_{\theta }.$$

The gradient of the objective function can be written as

(17) $$\nabla_{\theta }D\left(\theta \right)=E_{T\sim \pi \theta }\sum_{t=0}\vartheta^{t}Q^{\pi }\left(q_{t},b_{t}\right)\nabla_{\theta }{\log\pi }_{\theta }\left(\frac{b_{t}}{q_{t}}\right).$$

**Figure 6:**
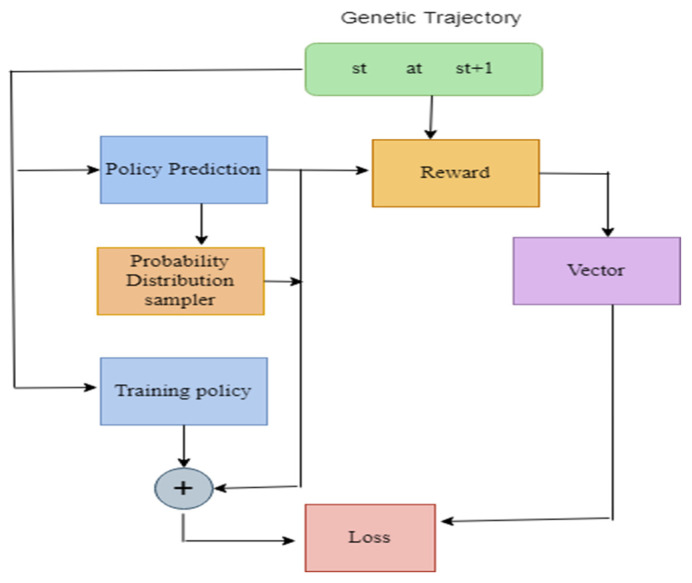
Framework of the Policy Gradient model.

Figure 6 illustrates the Framework of the Policy Gradient model. Here, the genetic trajectory consists of st up to st+1. It is connected to the policy prediction and the reward; the set of functions are loaded in the policy prediction. The sample values are calculated based on the probability distribution. The vectors values are generated from the reward. The loss function helps to generate the loss value; the gradient values are updated by backpropagating the network.

##### Actor–Critic

Actor–Critic executes the policy gradient with the help of value-based function. The concept of Actor–Critic is to divide the model into two parts: (i) executes an action depends on state; and (ii) generates the q value. The advantage of the Actor–Critic network is that it consists of two networks, namely, actor network and critic network.

(18) $$\;D_{\pi }^{\theta }=E_{T\emptyset }\sum_{t=0}\log(\pi_{\theta }\left(\frac{b_{t}}{q_{t}}\right)\vartheta^{t}$$

(19) $$D^{\pi }\left(q_{t},b_{t}\right)-{\mathrm{val}}_{t}^{\pi }$$

It can be written as

(20) $$Z\left[q_{t-1},q_{t}\right]-{\mathrm{val}}_{t+1}^{\pi }-\vartheta_{t}^{\pi }.$$

**Figure 7:**
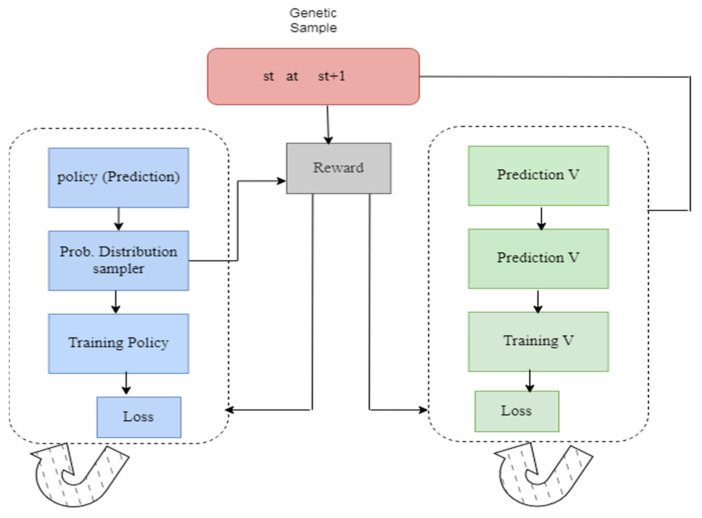
Framework of the Actor–Critic model.

Figure 7 illustrates the framework of the Actor–Critic model. Initially, the policy is predicted, then the probability distribution function is given; later, the process leads to training policy and the loss value are measured. The model is repeated until the loss value reduced. The loss value should be as low as possible; the model is executed repeatedly until the loss value becomes sufficiently low. Later, the model is moved to the next phase; here, the models are predicted and trained accordingly.

**Figure 8:**
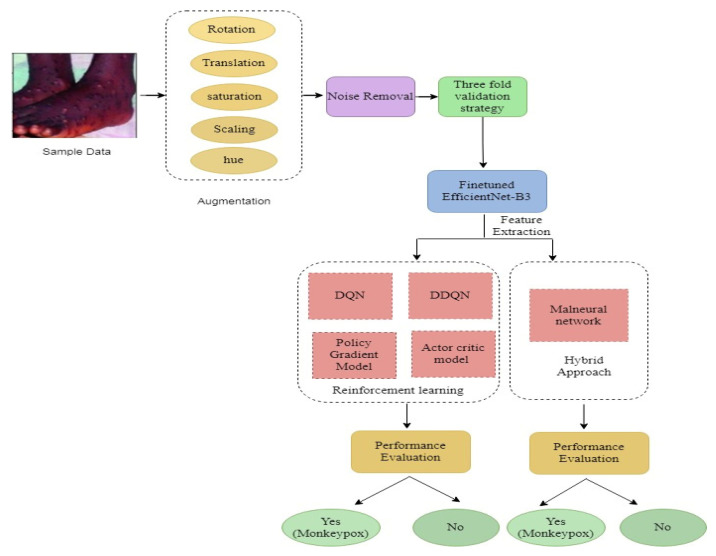
Proposed system framework.

Figure 8 illustrates the proposed framework. Initially, the “Monkeypox Image Lesion Dataset” was produced with the primary goal of separating monkeypox patients from related non-monkeypox instances. As a consequence, to conduct classifier, we introduced lesion pictures of “Chickenpox” and “Measles” to the “Monkeypox” category because of its similarity to the monkeypox rash and the initial state pustules. It has a maximum of 228 photos, of which 102 are under the “Monkeypox” label and the remaining 126 under the “Others” label, which include cases of non-monkeypox. The count of the dataset is enhanced further by using image enhancement techniques, including rotation, translation, reflection, shear, hue, saturation, contrast and brightness jitter, noise, scaling, etc. These were implemented with the help of MATLAB R2020a to help with the classification problem. After augmentation, the numbers of images were 1428 and 1764, respectively, in the classifications “Monkeypox” and “Others”. The next process is three-fold cross validation; it was carried out in order to remove any bias from the training process. The dataset was divided about 70:10:20 into training, validation, and test sets. The next process is feature extraction; the given features are extracted using fine-tuned Efficient-B3. Once the features are extracted, they proceed to the next phase, the classification phase. The images are classified by using two different approaches, namely, the reinforcement learning approach and the hybrid approach. In the first approach, the individual methods, namely DQN, DDQN, Policy Gradient, and the Actor–Critic Model, are applied over the extracted features. In the second approach Algorithm 1, the hybrid model called the Malneural network is developed. In this approach, the deep neural Q-learning and Policy Gradient models are tuned.
**Algorithm 1:** Malsneural algorithm 
1. Procedure Augmentation(image, pro) 
2.    prob pro: 
3.                  image←Rotate (image,(−5, +5))

4.    prob pro: 
5.            image←Translate (image,(0.8, 1.2))

6.    prob pro: 
7.            image←Saturation(image)

8.    prob pro: 
9.             image←Scaling(image,(0.7, 1.2))

10.    prob pro: 
11.             image←hue(image)

12. Return image 
13. Adaptive median filter 
14. Level 1: 
15. $\;\;\;\;\;\;\;\;image\;1=Z_{median}-Z_{min}$

16. $\;\;\;\;\;\;\;\;image\;2=Z_{median}-Z_{max}$

17.     If image1 > 0 and image 2 < 0 go to the next level 
18.     Else the size of the window increased 
19.        If windoe size <= size max redo the level 1 
20.        Else return zxy 
21. Level 2: 
22. $\;\;\;\;\;\;\;\;\;\;\;\;\;image\;3=Z_{xy}-Z_{min}$

23. $\;\;\;\;\;\;\;image\;4=Z_{xy}-Z_{max}$

24.     If image 3 > 0 and image 4 < 0 return zxy 
25.     Else return zmedian 
26.     End if 
27. Load replay memory M to the capacity C 
28. Load the function action Q along with arbitrary weight W 
29. Load destination value function Q along with weight W- = W 
30. For iteration = 1,N do 
31.      Load sequence t = {y1} and preprocessed ϕ1 = ϕ(t1) 
32.  For q = 1, Q do 
33.      The random action choosen bQ 
34.      Orelse choose bq = argmaxb P(ϕ(tq),b;W) 
35.      Compile bq in emulator and notice reward rq and yq + 1 of input 
36.      Set t q + 1 = tq, bq, y q + 1 and process ϕq + 1 = ϕ(tq + 1) 
37.      Save the transition (ϕq, bq,rq,ϕq + 1) in M 
38.      Minibatch (ϕi, bi, fi,ϕi + 1 ) from M 
39.      If it stops at i + 1 
40.      Initialise fj 
41.      Else 
42.      Yj = {fi + ϑmax d P(ϕi + 1,bq,W) 
43.      Execute gradient descent by updating the gradient value (yi-P(ϕi,bi; W))2 
44.      Reset ό = P 
45.  End for 
46. End for 

## 4. Experiment and Analysis

### 4.1. Experimental Setup

In this section, the experimental analysis is discussed. Initially, the fine-tuned EfficientNet B3 model was build and executed. The fine-tuned layers are listed below, and add a 0.5 dropout layer. The reason to add drop out is to reduce the overfitting problem. One flattened layer, two dense, and two dropout layers are added. The fine-tuned model reduces the model generalization problem. The parameters include the follows: the optimizer is the Adam optimizer, the learning rate set to 0.001, the loss value is set to Binary cross entropy, and the epoch value set to 200, along with batch size 32 as represented in the Table 3.

#### 4.1.1. Precision

Precision asks the question of what percentage of all the optimistic predictions is genuinely positive.

(21)           precision=True positiveTrue positive+False positive

The precision value lies between 0 and 1.

#### 4.1.2. Recall

Recall states the proportion of the total is anticipated to be positive.

(22) Recall=True positiveTrue positive+False negative 

#### 4.1.3. F1 Score

F1 Score combination of precision and recall. It takes both false positives and false negatives into account. As a result, it performs well with a dataset that is unbalanced.

(23) F1score=2∗(Precision∗Recall)(Precision+Recall)

#### 4.1.4. Recall and F1 Score Are given Equal Weighted values

There is a weighted F1 score that allows us to assign different weights to recall and precision. Recall and precision are assigned different weights in different issues, as described in the previous section.

(24)  Fβ=(1+β2)∗(Precision∗Recall)(β2∗Precision)+Recall

Beta is the number of times recall is more important than precision. If recall is twice as important as precision, the value of Beta is 2.

**Figure 9:**
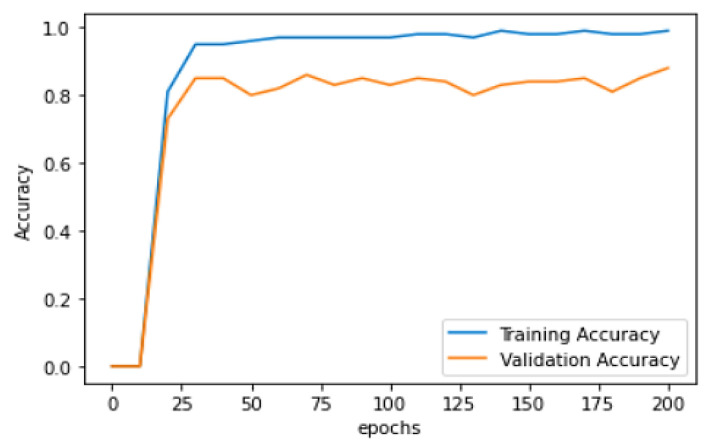
Training and validation learning curve.

Table 4 represents the training and validation performance to epoch count. In this work, the model is executed up to epoch 200. Initially, at epoch 10, the training accuracy was 0.817, training loss was 1.0625, validation accuracy was 0.738, and validation loss was 0.880. The performance gradually increases in every epoch count. The training accuracy keeps on increasing and testing loss keeps on decreasing. Finally, at the epoch 200, the model obtained a training accuracy of 0.9907, training loss of 0.0528, validation accuracy of 0.8571, and validation loss of 0.9906. Figure 9 represents the learning curve of training and validation accuracy; in the learning curve, the generalization gap does not increase. The training and validation learning curve decreases at a point of stability.

**Figure 10:**
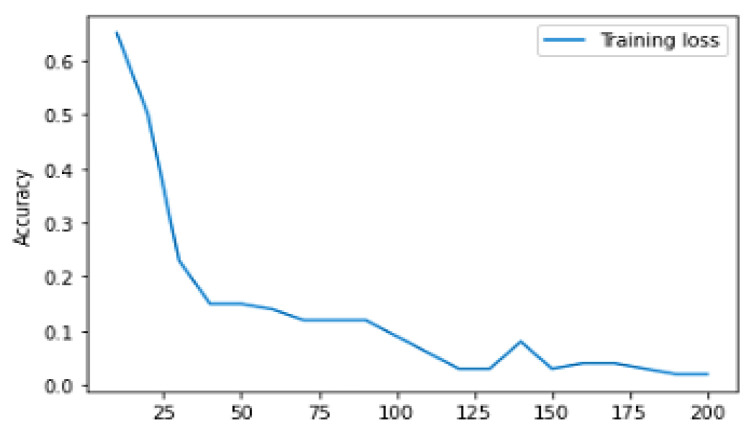
Training loss learning curve.

**Figure 11:**
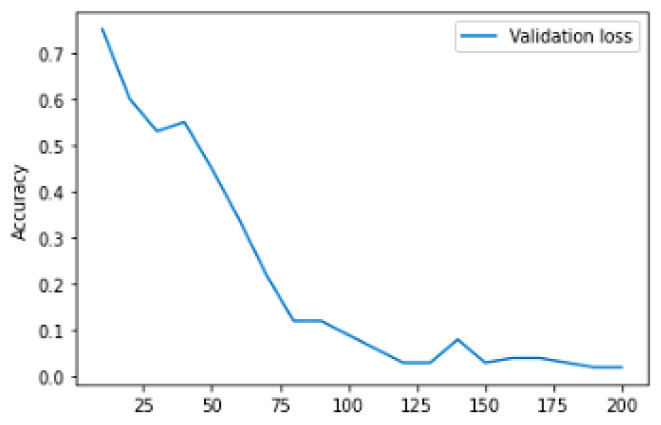
Validation loss learning curve.

Figure 9 represents Training and validation learning curve and Figure 10 represents the learning curve of training loss; in the learning curve, the generalization gap does not increase; the training and validation learning curve decreases at a point of stability. Figure 11 represents the validation loss learning curve; the learning curve keeps on decreasing and attains the stable value.

**Figure 12:**
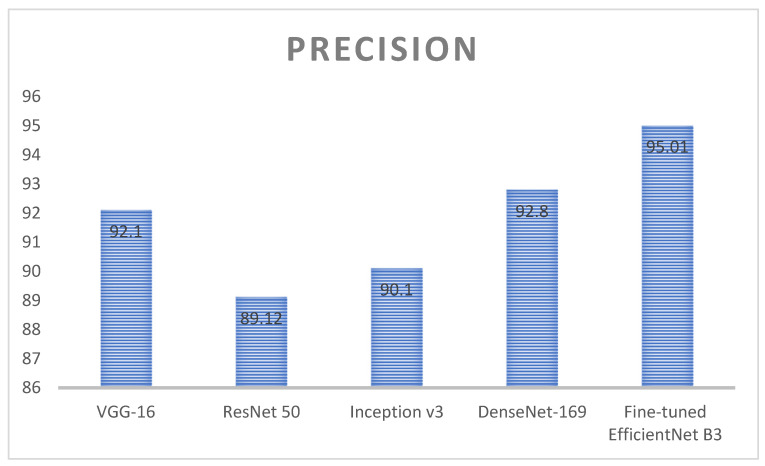
Analysis of precision value for the deep learning algorithms.

Figure 12 represents the analysis of precision value for deep learning algorithms; here, four different algorithms are taken, namely, VGG-16, ResNet 50, inception v3, and DenseNet 169. These algorithms are kept as a benchmark and compared with the proposed method called fine-tuned EfficientNet B3. VGG 16 obtained a precision value around 92.1%, ResNet 50 obtained a precision value around 89.12%, Inception v3 obtained a precision value around 90.1, and DenseNet 169 obtained a precision value around 92.8%. Here, the proposed method obtained a higher accuracy (around 95.01), which is higher compared with the remaining approach. Table 5 represents the performance evaluation of monkeypox detection.

**Figure 13:**
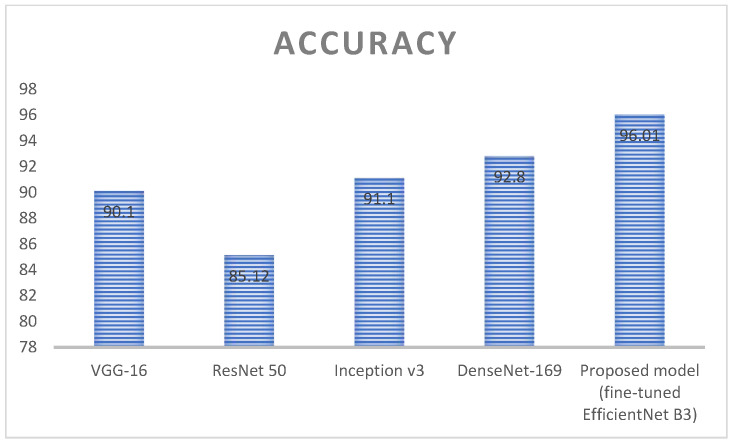
Analysis of accuracy value for the deep learning algorithms.

Figure 13 represents the analysis of accuracy value for deep learning algorithms; here, four different algorithms are taken, namely VGG-16, ResNet 50, inception v3, and DenseNet 169. These algorithms are kept as a benchmark and compared with the proposed method called fine-tuned EfficientNet B3. VGG 16 obtained an accuracy value around 90.1%, ResNet 50 obtained an accuracy value around 85.12%, Inception v3 obtained an accuracy value around 91.1, and DenseNet 169 obtained an accuracy value around 92.8%. Here, the proposed method obtained a higher accuracy (around 96.01), which is higher compared with the remaining approach. Table 6 represents the performance evaluation of monkeypox detection.

**Figure 14:**
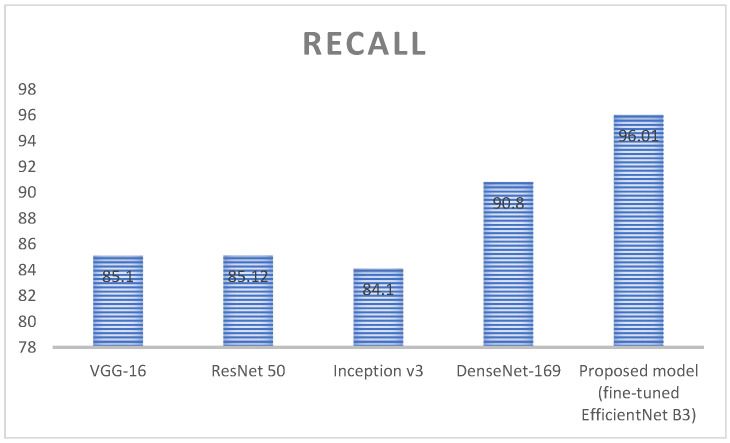
Analysis of Recall value for the deep learning algorithms.

Figure 14 represents the analysis of recall value for deep learning algorithms; here, four different algorithms are taken, namely, VGG-16, ResNet 50, inception v3, and DenseNet 169. These algorithms are kept as a benchmark and compared with the proposed method called fine-tuned EfficientNet B3. VGG 16 obtained a recall value around 85.1%, ResNet 50 obtained a recall value around 85.12%, Inception v3 obtained a recall value around 84.1, and DenseNet 169 obtained a recall value around 90.8%. Here, the proposed method obtained a higher accuracy (around 96.01), which is higher compared with the remaining approach. Table 7 represents the performance evaluation of monkeypox detection.

**Figure 15:**
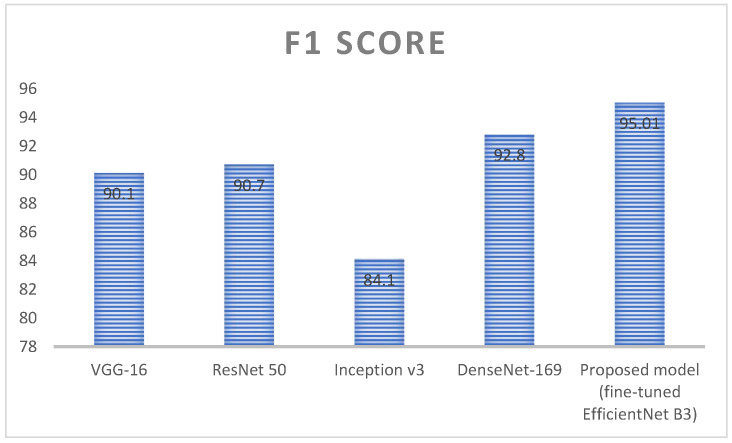
Analysis of F1 score value for the deep learning algorithms.

Figure 15 represents the analysis of F1 score value for deep learning algorithms; here, four different algorithms are taken, namely, VGG-16, ResNet 50, inception v3, and DenseNet 169. These algorithms are kept as a benchmark and compared with the proposed method called fine-tuned EfficientNet B3. VGG 16 obtained an F1 score value around 90.1%, ResNet 50 obtained an F1 score value around 90.7%, Inception v3 obtained an F1 score value around 84.1, and DenseNet 169 obtained an F1 score value around 92.8%. Here the proposed method obtained a higher accuracy (around 95.01), which is higher compared with the remaining approach. Table 8 represents the performance evaluation of monkeypox detection.

**Figure 16:**
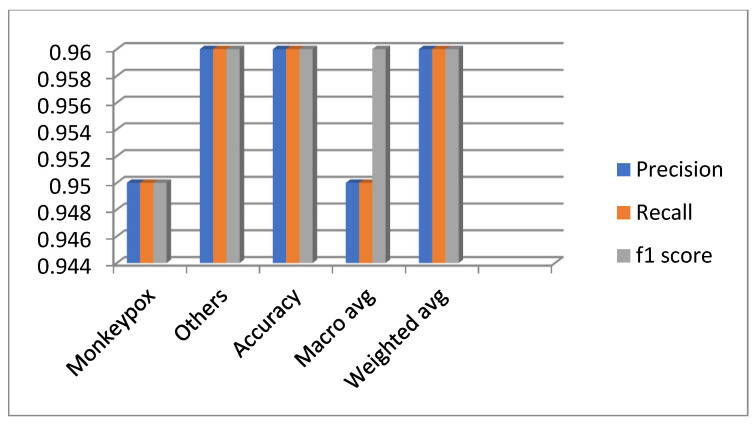
Overall performance evaluation of the diseases.

Figure 16 states that for the monkeypox class the precision value achieved around 0.95, recall value achieved around 0.95, and f1 score value achieved around 0.95. For other classes the precision value achieved around 0.96, recall value achieved around 0.96, and f1 score value achieved around 0.96. The overall macro average achieved around 0.95 and overall weighted average is 0.96.

**Figure 17:**
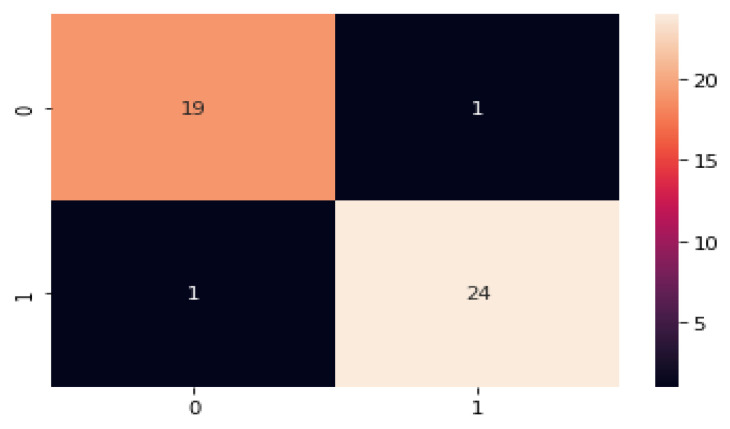
Confusion matrix for monkeypox disease classification.

Figure 17 represents the confusion matrix for the monkeypox disease detection; the values are generated based on the true positive, true negative, false positive, and false negative. Here most of the classes are recognized correctly and performs better.

**Figure 18:**
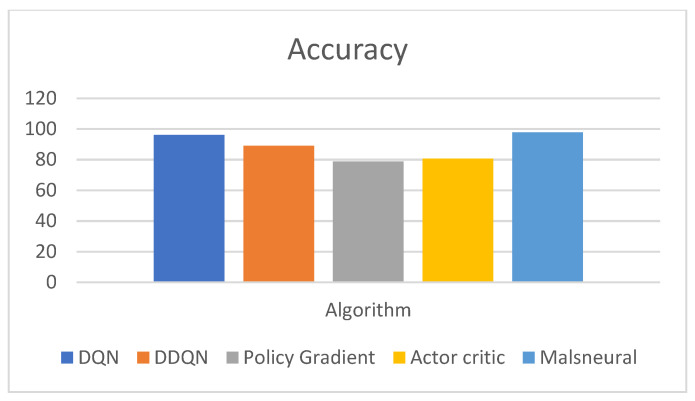
Analysis of Reinforcement learning algorithm for the classification of monkeypox.

Figure 18 represents the analysis of accuracy value for reinforcement learning algorithms; here, four different algorithms are taken, namely DQN, DDQN, Policy Gradient, and Actor–Critic. These algorithms are kept as a benchmark and compared with the proposed method called Malneural. DQN obtained an accuracy value around 96.5%, DDQN obtained an accuracy value around 89.7%, Policy Gradient obtained an accuracy value around 78.7%, and Actor–Critic obtained an accuracy value around 80.7%. Here, the proposed method obtained a higher accuracy (around 97.7%), which is higher as compared with the remaining approach. Table 9 represents accuracy calculation for the monkeypox disease detection results using the reinforcement learning approach.

**Figure 19:**
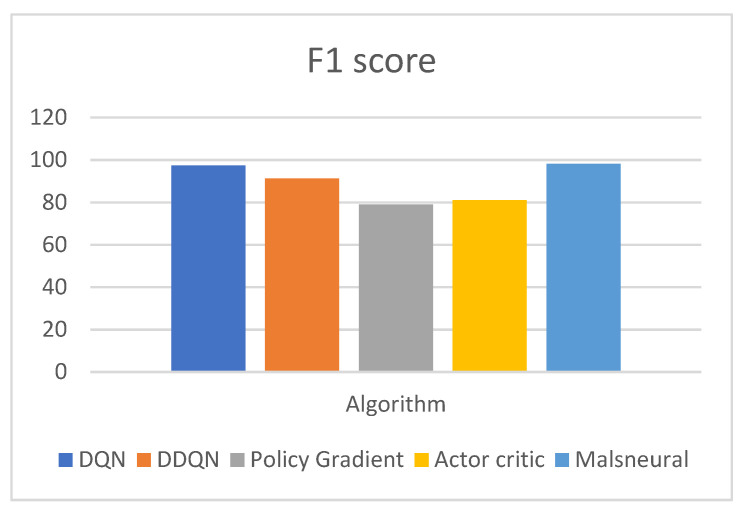
Analysis of reinforcement learning algorithm for the classification of monkeypox.

Figure 19 represents the analysis of f1 score value for reinforcement learning algorithms; here, four different algorithms are taken, namely, DQN, DDQN, Policy Gradient, and Actor–Critic. These algorithms are kept as a benchmark and compared with the proposed method called Malneural. DQN obtained an f1 score value around 97.4%, DDQN obtained an f1 score value around 91.2%, Policy Gradient obtained an f1 score value around 79.0%, and Actor–Critic obtained an f1 score value around 81.1%. Here, the proposed method obtained a higher accuracy (around 98.1%), which is higher as compared with the remaining approach. Table 10 represents the accuracy calculation monkeypox disease detection results using the reinforcement learning approach.

**Figure 20:**
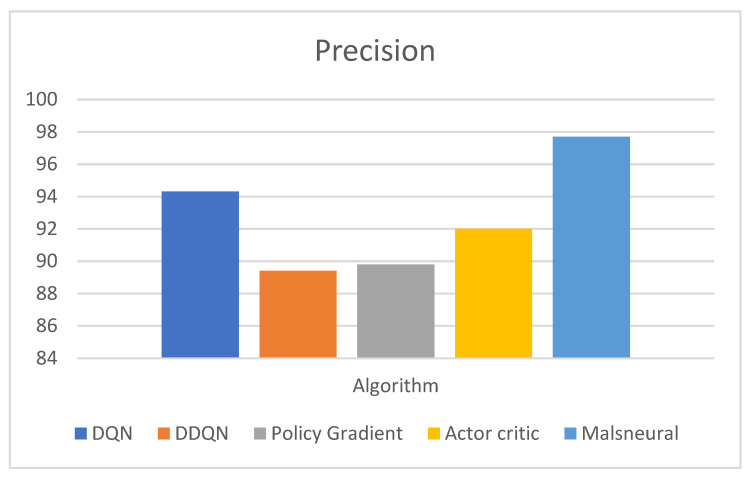
Analysis of reinforcement learning algorithm for the classification of monkeypox.

Figure 20 represents the analysis of precision value for reinforcement learning algorithms; here, four different algorithms are taken, namely DQN, DDQN, Policy Gradient, and Actor–Critic. These algorithms are kept as a benchmark and compared with the proposed method called Malneural. DQN obtained a precision value around 94.3%, DDQN obtained a precision value around 89.4%, Policy Gradient obtained a precision value around 89.4%, and Actor–Critic obtained a precision value around 92.0%. Here the proposed method obtained a higher accuracy (around 96.1%), which is higher compared with the remaining approach. Table 11 represents the accuracy calculation for monkeypox disease detection results using the reinforcement learning approach.

**Figure 21:**
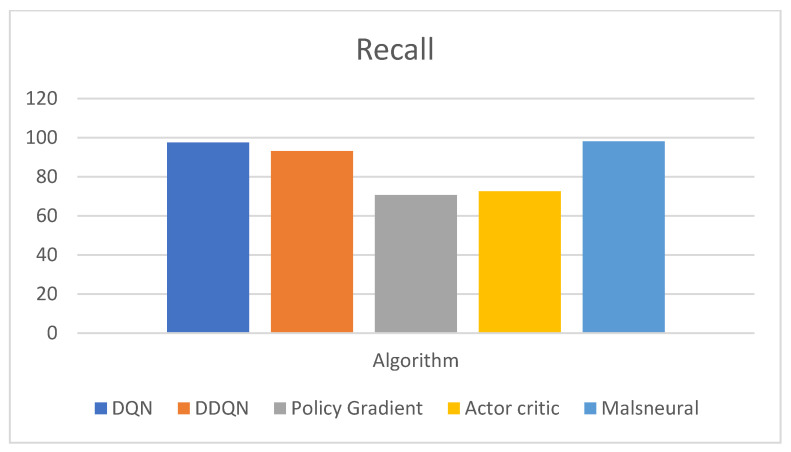
Analysis of reinforcement learning algorithm for the classification of monkeypox.

Figure 21 represents the analysis of precision value for reinforcement learning algorithms; here, four different algorithms are taken, namely, DQN, DDQN, Policy Gradient, and Actor–Critic. These algorithms are kept as a benchmark and compared with the proposed method called Malneural. DQN obtained an f1 score value around 97.4%, DDQN obtained an f1 score value around 93.0%, Policy Gradient obtained an f1 score value around 70.6%, and Actor–Critic obtained an f1 score value around 72.5%. Here, the proposed method obtained a higher accuracy (around 98.1%), which is higher compared with the remaining approach. Table 12 represents the accuracy calculation monkeypox disease detection results using the reinforcement learning approach.

## 5. Conclusions and Future Scope

In this work, the classification of monkeypox diseases was identified. Initially, a fine-tuned EfficientNet B3 model was built and executed. The fine-tuned layers includes the 0.5 dropout layer. The reason to add the dropout layer is to reduce the overfitting problem. One flattened layer, two dense, and two dropout layers are added. The fine-tuned model reduces the model generalization problem. The parameters include the following: the optimizer is the Adam optimizer, the learning rate set to 0.001, loss value is set to Binary cross entropy, and the epoch value set to 200, along with a batch size of 32. The model was compared with the reinforcement learning approach, namely, DQN, DDQN, Policy Gradient, and Actor–Critic. The resultant analysis demonstrates that DQN obtained the highest accuracy (around 0.975). For the monkeypox class, the precision value achieved around 0.95, the recall value achieved around 0.95, and the f1 score was around 0.95. For other classes, the precision value achieved around 0.96, the recall value achieved around 0.96, and the f1 score value was around 0.96. The overall macro average achieved around 0.95 and the overall weighted average was 0.96. It is envisaged that transfer learning models will be developed on this dataset in the future and will perform better than the present CNN models. We also plan to train the models described in the research with bigger datasets as well. It is also anticipated that generative adversarial network (GAN)-based CNN models will be developed and evaluated against the current models. Future work will incorporate this model in clinics and hospitals.

## Figures and Tables

**Table 1:** Data analysis for detection of monkeypox disease detection for feature extraction model.

Reference	Techniques	Datasetcount	Recall (%)	Accuracy (%)	F1 Score (%)	Precision (%)
[34]	VGG-16	1428	81.0	81.48	83.01	85.01
[34]	ResNet 50	1428	83.0	82.96	84.01	87
[34]	Inception v3	1428	81.0	74.07	78	74.10
[35]	DenseNet-169	1784	83.00	84.24	83.83	83.12

**Table 2:** Data analysis for the detection of monkeypox disease detection for the classification process.

Reference	Techniques	Datasetcount	Precision (%)	Accuracy (%)	Recall (%)	F1 Score (%)
[34]	DQN	1428	84	79.26	79.0	81.1
[36]	DDQN	1000	79.2	84.0	79.0	81.0
[37]	Policy Gradient	1200	80.8	85.1	91.1	76.5
[38]	Actor–Critic	89.0	63.0	92.0	74.0	90.0

**Table 3:** Fine-tuned hyperparameters.

Parameters	Values
Optimizer	Adam
Learning rate	0.001
Loss	Binary_crossentropy
epoch	200
Batch size	32

**Table 4:** Performance evaluation of fine-tuned EfficientNet B3 with respect to accuracy and loss.

Epoch	Training Accuracy	Training Loss	Validation Accuracy	Validation Loss
10	0.8179	1.0625	0.7381	0.8807
20	0.9519	0.3025	0.8524	0.6117
30	0.9534	0.2310	0.8095	1.0032
40	0.9631	0.1515	0.7881	1.4253
50	0.9701	0.1502	0.7238	1.7884
60	0.9720	0.1743	0.9626	0.8833
70	0.9757	0.1239	0.8381	1.1028
80	0.9753	0.1242	0.8595	0.9159
90	0.9795	0.1258	0.8310	1.3588
100	0.9823	0.0971	0.8524	0.9706
110	0.9841	0.0676	0.8405	1.5409
120	0.9771	0.1351	0.8095	1.4022
140	0.9925	0.0320	0.8310	1.3769
150	0.9851	0.0806	0.8429	1.2375
160	0.9893	0.0393	0.8405	1.0522
170	0.9916	0.0394	0.8476	1.1742
180	0.9874	0.0470	0.8571	1.5278
190	0.9897	0.0476	0.8119	1.1780
200	0.9907	0.0528	0.8571	0.9906

**Table 5:** Precision calculation for detection of monkeypox disease detection.

Techniques	Precision (%)	Dataset Count
VGG-16	92.1	3192
ResNet 50	89.12	
Inception v3	90.1	
DenseNet-169	92.8	
Proposed model (fine-tuned EfficientNet B3)	95.01	

**Table 6:** Accuracy calculation for detection of monkeypox disease detection.

Techniques	Accuracy (%)	Dataset Count
VGG-16	90.1	3192
ResNet 50	85.12	
Inception v3	91.1	
DenseNet-169	92.8	
Proposed model (fine-tuned EfficientNet B3)	96.01	

**Table 7:** Recall calculation for detection of monkeypox disease detection.

Techniques	Recall (%)	Dataset Count
VGG-16	85.1	3192
ResNet 50	85.12	
Inception v3	84.1	
DenseNet-169	90.8	
Proposed model (fine-tuned EfficientNet B3)	96.01	

**Table 8:** F1 score calculation for detection of monkeypox disease detection.

Techniques	F1 Score (%)	Dataset Count
VGG-16	90.1	3192
ResNet 50	91.12	
Inception v3	84.1	
DenseNet-169	90.7	
Proposed model (fine-tuned EfficientNet B3)	95.01	

**Table 9:** Accuracy calculation for monkeypox disease detection results using the reinforcement learning approach.

Reinforcement Learning	Model	Accuracy
	DQN	96.5
	DDQN	89.7
	Policy Gradient	78.7
	Actor–Critic	80.7
	Malneural	97.7

**Table 10:** F1 score calculation for detection of monkeypox disease detection results using the reinforcement learning approach.

Reinforcement Learning	Model	F1 Score
	DQN	97.4
	DDQN	91.2
	Policy Gradient	79.0
	Actor–Critic	81.1
	Malneural	98.1

**Table 11:** Precision calculation for detection of monkeypox disease detection results using the einforcement learning approach.

Reinforcement Learning	Model	Precision
	DQN	94.3
	DDQN	89.4
	Policy Gradient	89.8
	Actor–Critic	92.0
	Malneural	96.1

**Table 12:** Recall calculation for monkeypox disease detection results using the reinforcement learning approach.

Reinforcement Learning	Model	Recall
	DQN	97.4
	DDQN	93.0
	Policy Gradient	70.6
	Actor–Critic	72.5
	Malneural	98.1

## Data Availability

Data will be available on request from first author.
